# Gold Nanorods Enable Rapid and Label-Free SARS-CoV‑2 Detection through LAMP Assay

**DOI:** 10.1021/acsomega.5c08097

**Published:** 2026-01-23

**Authors:** Amanda Bonoto Gonçalves, Iara Apolinário Borges, Kennedy Batista Gonçalves, Caroline Magalhães Junqueira, Letícia Trindade Almeida, Rosimeire Coura Barcelos, Pedro Augusto Alves, Lívia Siman Gomes, Rubens Lima do Monte-Neto, Anna Carolina Pinheiro Lage

**Affiliations:** † Grupo de Pesquisas em Biotecnologia Aplicada ao Estudo de Patógenos (BAP) - Instituto René Rachou, Fundação Oswaldo Cruz, IRR/Fiocruz Minas, Av. Augusto de Lima, 1715, Belo Horizonte 30190-009, Minas Gerais, Brazil; ‡ Departamento de Física, Instituto de Ciências Exatas, Universidade Federal de Minas Gerais, Av. Presidente Antônio Carlos 6627, Pampulha, 31270-901 Belo Horizonte, Brazil; § Centro de Tecnologia em Nanomateriais e Grafeno da Universidade Federal de Minas Gerais, CTNano − UFMG, Rua Professor José Vieira de Mendonça 520, Engenho Nogueira, 31310-260 Belo Horizonte, Brazil; ∥ Universidade Federal De São João Del Rei, Campus Centro Oeste Dona Lindu, Rua Sebastião Gonçalves Coelho, 400, Chanadour, 35501-296 Divinópolis/MG, Brazil

## Abstract

Nanoscale noble metals exhibit unique optical properties due to localized surface plasmon resonance (LSPR), which occurs within the visible range of the electromagnetic spectrum. This property has facilitated their extensive use as transducers in biosensor platforms. Among these, gold nanoparticles—particularly gold nanorods (AuNRs)—have gained significant attention owing to their superior plasmonic characteristics. Their anisotropic shape generates two distinct plasmonic bands, enhancing light absorption, scattering, and sensitivity to environmental changes. Here, we present a biosensing approach for detecting the SARS-CoV-2 genetic material amplified via loop-mediated isothermal amplification (LAMP), using AuNRs as transducers to generate dynamic light scattering signals captured by a custom-built depolarized dynamic light scattering (DDLS) apparatus. This strategy reduces the conventional LAMP reaction time to 20 min and allows direct detection of target amplicons, representing a substantial improvement over traditional indirect detection methods that are often prone to false results. Clear discrimination between negative and positive samples was achieved using both control reactions and clinical specimens, demonstrating the potential of integrating AuNR-based transducers with LAMP and DDLS into an efficient, rapid, and highly sensitive diagnostic platform.

## Graphical abstract

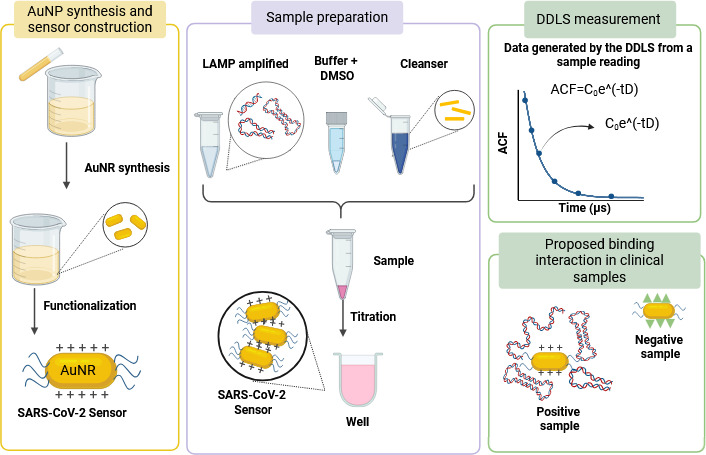

## 1 Introduction

The use of gold nanorods (AuNRs) as biosensors holds significant potential for label-free detection through techniques that monitor changes in localized surface plasmon resonance (LSPR).^1,2^ AuNRs are particularly attractive for diagnostic applications due to their scalable synthesis, versatile surface functionalization, and unique physicochemical and optical properties that enable studies involving maximum field enhancement at their tips. By functionalizing AuNRs with biomolecules – such as antibodies or molecular probes – and exposing them to target analytes, specific molecular interaction can be detected through shifts in the longitudinal LSPR peak in UV–vis spectra.^1–3^

Dynamic light scattering (DLS) is a widely used technique for size analysis of biomolecules and particulate materials, including gold nanoparticles.^4–8^ However, conventional DLS systems typically assume that particles are spherical, which can lead to significant inaccuracies when analyzing anisotropic nanostructures. In this study, we introduce a custom-built DDLS designed for biosensing applications using gold nanorods (GNRs) as anisotropic transducers.^9^ Unlike spherical particles, anisotropic GNRs depolarize scattered light due to their asymmetric geometry. The custom-built DDLS apparatus offers a simplified set up when compared to conventional instruments: it comprises a 650 nm laser source, optical lenses, polarizers and a CMOS sensor that collects light scattered by the GNRs. Acting as a multielement detector array, the CMOS sensor enables simultaneous acquisition of multiple light intensity scattering signals in a single measurement, thereby improving the signal-to-noise ratio. Light intensity autocorrelation functions (ACF) are processed on the camera board and its analysis directly returns, within seconds, information over the diffusion coefficient (*D*) of the scatters. GNR’s diffusion coefficient is highly sensitive to size alterations and thus, very interesting for biosensing application.^10–12^

LAMP technique is a nucleic acid amplification method that uses a set of primers to specifically recognize and amplify target regions in DNA or RNA sequences. Due to its unique amplification mechanism, LAMP generates significantly larger amounts of amplified genetic material than conventional PCR, and it operates at a constant temperature – eliminating the need for thermal cycling.^13^ This feature makes LAMP particularly suitable for point-of-care diagnostics and use in resource-limited settings.^14,15^ Despite its advantages, LAMP also has some limitations. A major concern is the reliance on indirect detection methods such as fluorometry, turbidimetry, fluorescence, or colorimetry using pH indicators. These approaches, while convenient, can increase the risk of false-positive results, thus compromising the reliability of LAMP-based diagnostics. Addressing this limitation is crucial to enhancing the accuracy and robustness of LAMP for clinical and field applications.^12–15^ A number of approaches have been proposed to address the occurrence of false-positive results in LAMP assays, reflecting ongoing efforts to enhance the specificity and robustness of this method.^16–19^

To improve the simplicity, reliability, sensitivity, and specificity of LAMP-based detections, this study introduces a novel diagnostic platform that integrates LAMP amplification with direct detection using AuNRs biosensors coupled to a DDLS device. This approach enables rapid detection of amplified DNA within 20 min, representing a significant advancement over conventional LAMP methods that rely on indirect readouts. The platform was initially applied to the detection of SARS-CoV-2, yielding highly promising results. This integrated system represents a potential technological breakthrough in molecular diagnostics, with considerable scope for further optimization and broad applicability across diverse diagnostic settings.

## 2 Materials and Methods

### 2.1 AuNR Synthesis

AuNRs were employed as biosensor transducers and synthesized following the method described by Wang et al. with some modifications.^20^ Briefly, a growth solution was prepared containing HAuCl_4_ (0.65 mM), AgNO_3_ (0.11 mM), CTAB (50 mM), and resveratrol (5 mM). To initiate AuNRs growth, 600 μL of freshly prepared NaBH_4_ solution (3.0 mM) was added, and the mixture was incubated at 70 °C for 6 h. The resulting suspension was centrifuged at 10,000 × *g* for 20 min, and the AuNRs-containing pellet was collected and resuspended in CTAB (5 mM). All solutions were prepared using ultrapure water (ρ (resistivity) = 18 mΩ·cm) from a Millipore Milli-Q system. The reagents including – including chloroauric acid (≥99.0%), CTAB (≥98.0%), silver nitrate (≥99.0%), sodium borohydride (96.0%), and ascorbic acid (AA) (≥99.0%) – were purchased from Sigma-Aldrich Chemical Co. (USA). Resveratrol was sourced as a pharmaceutical-grade input imported from China by Fagron BR.

### 2.2 AuNR Physicochemical Characterizations

The characterization of AuNRs was conducted using complementary techniques. UV–vis absorption spectroscopy (Thermo Scientific Varioskan LUX, 400–900 nm) was used to obtain the characteristic spectrum, with O.D. at 400 nm applied to estimate elemental gold concentration.^21,22^ The longitudinal LSPR peak position was employed to determine the aspect ratio (A.R.) of AuNRs,^23^ as this method is rapid, nondestructive, and statistically robust, reflecting the average colloidal suspension. Notably, the A.R. values obtained were consistent with TEM measurements, reinforcing its validity and in agreement with previous reports.^21^ Size was measured using the portable DDLS device developed at the Biophysics Laboratory, Physics Department, UFMG (Belo Horizonte, Brazil), and morphology confirmed by TEM (Tecnai G2–12 Spirit Biotwin, FEI, 120 kV).

### 2.3 AuNRs Biosensor Assembly and Characterization

The biosensing element of the sensor consisted of an oligonucleotide sequence, hereafter referred to as a probe, designed using SnapGene software. The probe was specifically engineered to hybridize within a region of the LAMP amplicon that does not overlap with the primer-binding sites, thereby minimizing nonspecific interaction. The probe sequence corresponds to a part of the SARS-CoV-2 N gene, which nucleotide sequence was 5′-S-AAAAAAAAAACTGGACTTCCCTATGGTGCTA-3′ and was synthesized by Exxtend. The functionalization medium consisted of CTAB (3 mM), TRIS buffer (10 mM), 75 μL of AuNRs (2 mM Au^0^), 15 μL of probe (100 pM/μL), and 1.5 μL of DTT (1 nM). The mixture was incubated under gentle magnetic stirring at room temperature overnight to allow efficient probe attachment to the AuNR surface. Following incubation, the samples were centrifuged at 3600 × *g* for 20 min. The pellets were resuspended in 1 mL of the same medium and subsequently analyzed using DDLS and UV–vis spectroscopy (Varioskan) to confirm successful biosensor assembly.

### 2.4 Reverse Transcription LAMP Assays

Reverse transcription LAMP (RT-LAMP) primers targeting the N gene of SARS-CoV-2 were designed using Primer Explorer V5, Eiken Corp. (https://primerexplorer.eiken.co.jp) and the New England Biolabs (NEB) LAMP primer design tool (https://lamp.neb.com), following the protocol described by Alves et al.^15^ Oligonucleotides were synthesized by Exxtend at a scale of 25 nmol and nucleotide sequences are listed in Supporting Information, Table S1. The RT-LAMP reaction mixture consisted of WarmStart Colorimetric LAMP 2× Master Mix (New England Biolabs), a complete set of inner, outer, and loop primers, and DNase/RNase-free ultrapure water. Positive controls included either inactivated SARS-CoV-2 RNA or synthetic plasmids encoding the N gene as templates. Reactions were terminated at intervals between 15 and 40 min. Amplification products were analyzed by gel electrophoresis, stained with GelRed, and visualized using the ImageQuant LAS 4000 system, confirming the specificity of the target amplification.

LAMP reaction products were evaluated using the biosensor platform with three types of reactions: a positive control (PC) containing SARS-CoV-2 RNA obtained from Vero cell cultures, a nontemplate control (NTC) using nuclease-free water, and clinical positive and negative test samples for proof-of-concept validation. Clinical RNA samples were generously provided by the Municipal Health Department of Belo Horizonte, Brazil. Nasopharyngeal swabs were collected from symptomatic hospitalized patients and preserved in viral transport medium (VTM) for subsequent RNA extraction. RT-qPCR confirmation of SARS-CoV-2 infection was conducted by the Health Department using the Allplex SARS-CoV-2 Assay (Seegene). All procedures involving human samples were approved by the appropriate institutional research ethics committee.

Quantification of amplified DNA during titration experiments was carried out using the Qubit dsDNA Broad Range (BR) Assay Kit. Specificity was evaluated through titration assays using pools of genetic material amplified by LAMP with pathogen-specific primer sets, including those targeting *Leishmania infantum* (Li) and Respiratory Syncytial Virus (RSV). Additionally, several breath-related pathogens previously tested with these primers confirmed the specificity of this assay for SARS-CoV-2 detection.

### 2.5 Confirming the Effectiveness of AuNRs as Biosensors for LAMP Assays by Different Reading Methods

Aliquots of the biosensor solution (300 μL) were dispensed into 96-well plates, followed by the addition of 20 μL from standard LAMP reactions conducted for 40 min. Initial assessments were performed through visual inspection of color changes and UV–vis absorption spectroscopy. For DDLS analysis, equal volumes of biosensor solution were distributed in the wells and titrated with varying volumes of pooled positive control (PC) and nontemplate control (NTC) LAMP reactions, halted at 15, 20, and 40 min. Prior to titration, each sample was diluted with 19 μL of reaction medium and 11 μL of ultrapure water. Plates were then incubated at 45 °C for 8 min, followed by a 5 min cooling phase at room temperature before analysis. This protocol enabled the detection of both biorecognition and nonbiorecognition events by the biosensor. Following the successful validation with control samples, clinical specimens were tested. To improve the accuracy of DDLS measurements, dimethyl sulfoxide (DMSO) and a proprietary cleanser were added to the biosensor medium.

## 3 Results

### 3.1 Synthesis and Characterizations of AuNRs

The synthesis of gold nanorods (AuNRs) yielded cylindrical nanoparticles, as shown in Figure 1a, with a characteristic UV–vis absorption spectrum depicted in Figure 1b. In colloidal dispersion, the AuNRs exhibited a distinct blue color observable to the naked eye (Figure 1c). DDLS analysis revealed a diffusivity (*D* ^–1^) of 10.0 ± 0.3 μs (Figure 1d), corresponding to a particle width of 33 nm. Comparison of this value with the aspect ratio (A.R.) derived from the UV–vis spectrum (Figure 1b) indicated an estimated diameter of 16 nm. These measurements were further validated by transmission electron microscopy (TEM), based on the average size of 100 individual particles. Spectral data obtained using the Varioskan system confirmed the localized surface plasmon resonance (LSPR) peak of AuNRs at 636 nm, in agreement with the excitation wavelength used in DDLS analysis.

**Figure 1:**
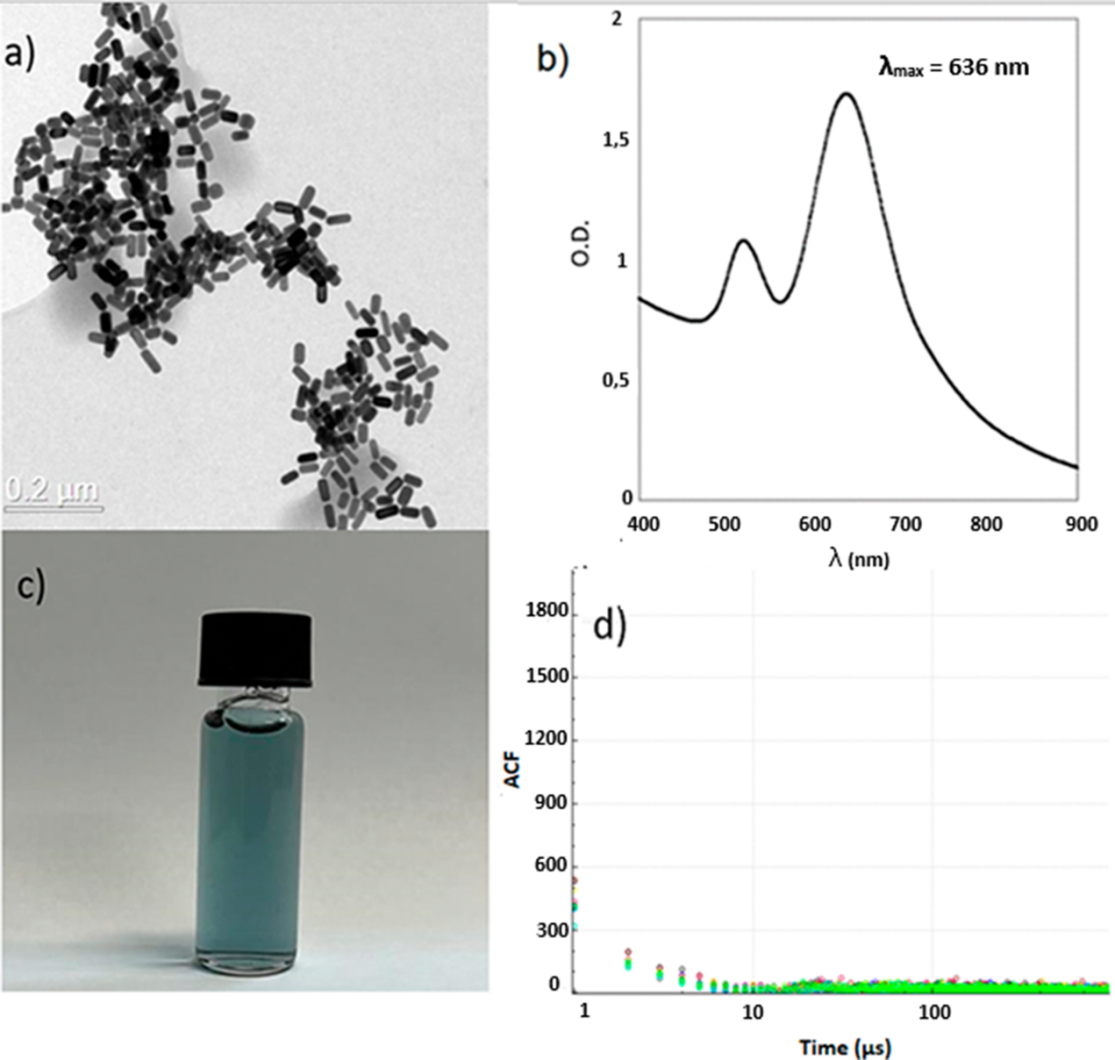
Synthesized AuNRs characterized by (a) TEM, (b) UV–vis absorption spectra in the scanning mode. (c) The particle formed a blue colloidal dispersion (D) with a *D* ^–1^ of 10.0 ± 0.3 μs as determined by DDLS using the exponential decay fitting of ACF. (d) The colors in the ACF versus time graph represent each of the ten individual measurements used in the *D* ^–1^ determination.

### 3.2 AuNR Biosensor Construction and Characterization

The UV–vis spectra of the bioconjugated AuNRs exhibited a pronounced red shift of 10.0 nm (Figure 2a), indicating a successful and confirming the formation of the biosensor through probe attachment to the AuNRs. Additionally, an increase of 5.0 μs in the *D* ^–1^ was observed (Figure 2b).

**Figure 2:**
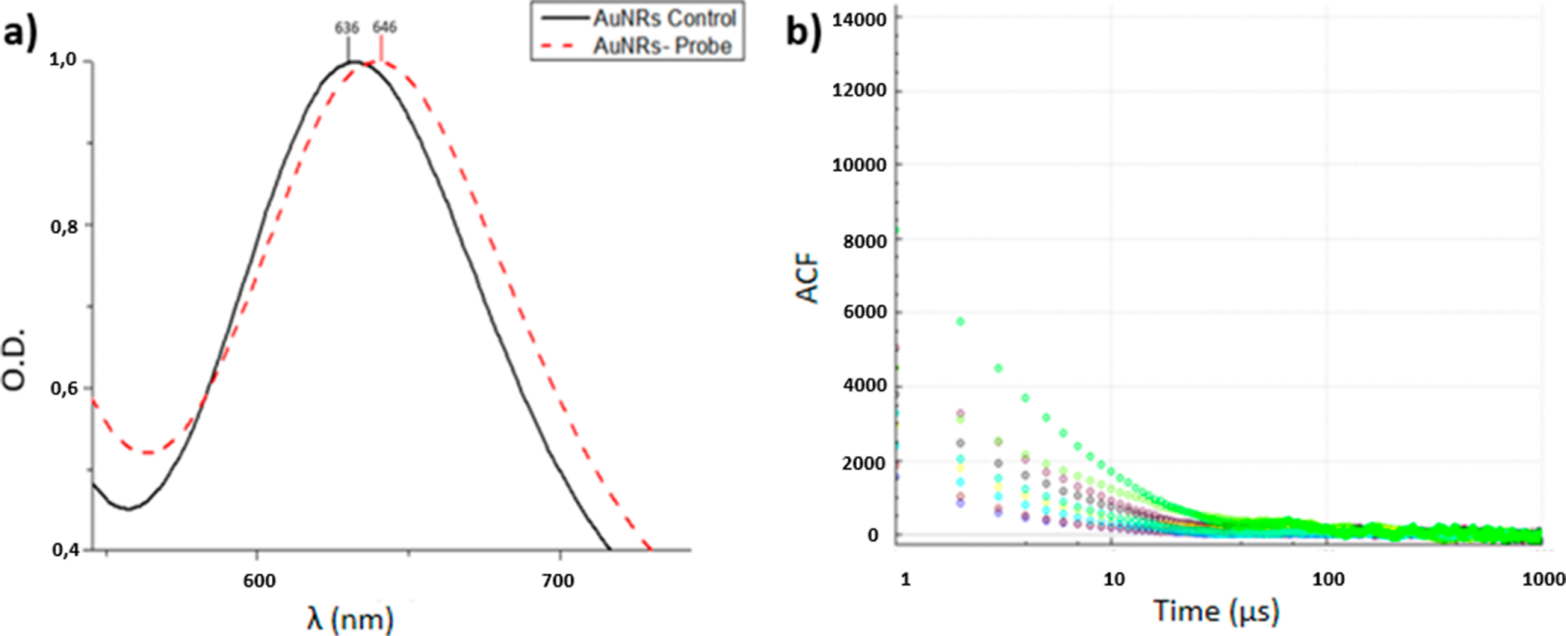
UV–vis characterization of nanosensors exposed to the positive and negative controls of SARS-CoV-2. (a) Detail of the red shift in the AuNs LSPR band following the probe binding process (b) and the corresponding increase in diffusivity (*D* ^–1^) from 10.0 ± 0.3 μs to 15.0 ± 0.5 as measured by the exponential decay fitting of ACF. The colors in the ACF versus time graph represent each of the ten individual measurements used in the *D* ^–1^ determination.

### 3.3 Reverse Transcription LAMP Assays

Figure 3 displays the colorimetric outputs and agarose gel electrophoresis of LAMP assays interrupted at 5 min intervals from 15 to 40 min. While the characteristic yellow color change, indicating a positive reaction, became visually evident after 25 min, electrophoresis revealed detectable amplification of SARS-CoV-2 genetic material as early as 20 min.

**Figure 3:**
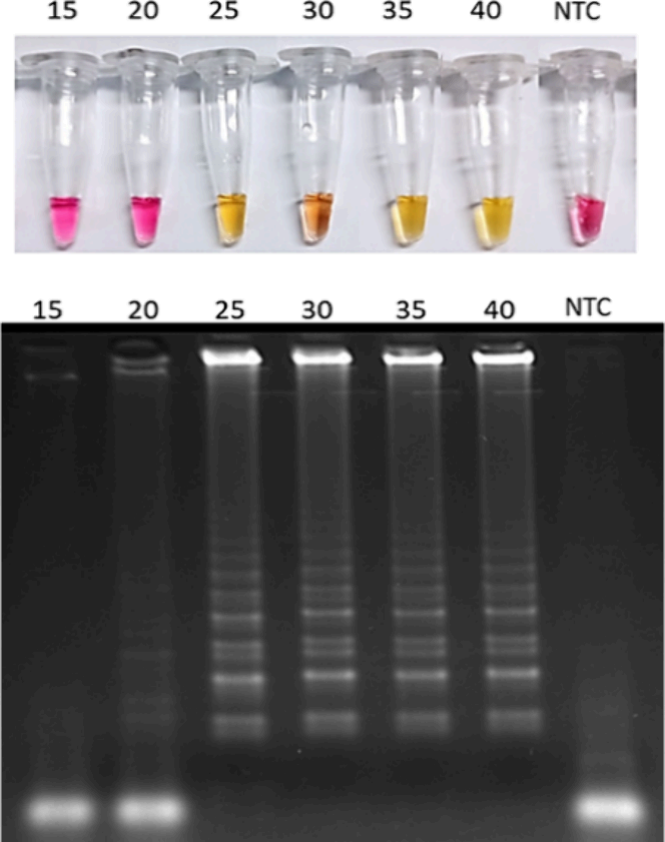
Colorimetric and gel electrophoresis output of LAMP reactions performed in different times. The reactions were interrupted at 15, 20, 25, 30, 35, and 40 min of reaction. The amplification products were resolved by agarose gel electrophoresis and analyzed in a transilluminator to confirm SARS-CoV-2 genetic material amplification. For positive reactions, we used the N gene plasmid (10 ng/μL). CP: positive control; NTC: nontemplate control.

### 3.4 Confirming Effectiveness of Biosensors for LAMP Assays by Different Reading Techniques

Biosensor performance was evaluated using a 96-well microplate, where each well was loaded with a 30 μL aliquot containing either LAMP positive control (PC) or nontemplate control (NTC) reactions. Distinctive colorimetric changes enabled straightforward visual interpretation. Wells containing PC samples consistently retained the characteristic blue color of the stable AuNRs dispersion. In contrast, NTC samples induced nanoparticle aggregation, leading to biosensor destabilization and a noticeable color shift from blue to light purple (top left of Figure 4). These visual findings were corroborated by UV–vis spectroscopy, which showed stable absorbance spectra for AuNRs exposed to PC, while spectra from NTC-exposed samples indicated partial nanoparticle disintegration (Figure 4).

**Figure 4:**
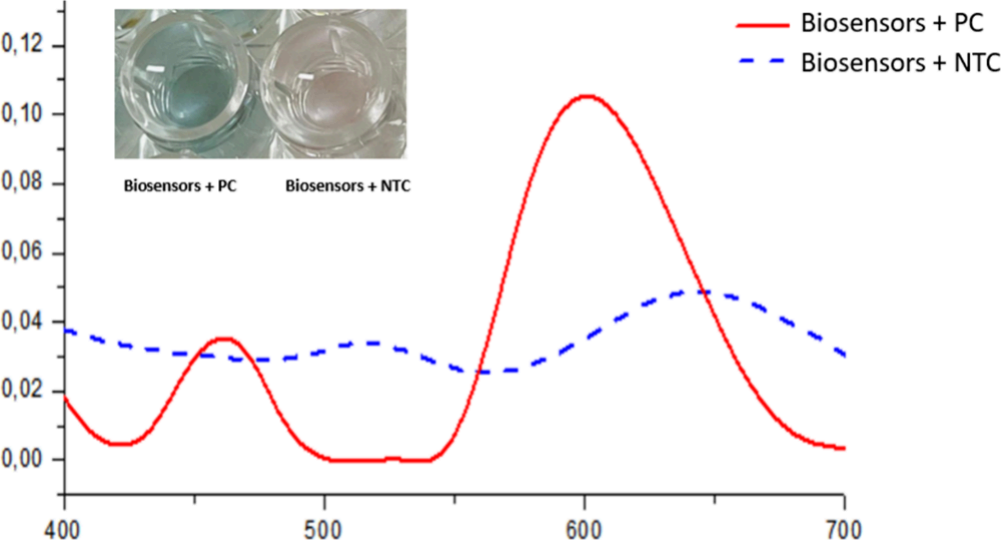
Biosensor response following titration with LAMP reaction products. The upper left panel shows that biosensors retain their blue color when exposed to positive control (PC) samples, whereas nontemplate control (NTC) samples induce a visible color shift, indicating nanoparticle aggregation. The accompanying absorbance spectra demonstrate spectral integrity for PC-treated samples, while NTC exposure results in spectral features consistent with AuNR disintegration. Phenol red background absorption was subtracted from the UV–vis spectra.

DDLS analysis of biosensor titrations with LAMP reaction products enabled the generation of graphical representations of diffusivity changes, expressed as Δ*D* ^–1^ (μs), which quantifies the shift in sensor diffusivity induced by analyte binding, relative to that of the unbound state. These values were calculated by subtracting the inverse diffusivity (*D* ^–1^) of the positive control (PC) from that of the nontemplate control (NTC), establishing a reference (Δ*D* ^–1^) to distinguish between positive and negative samples. When LAMP reactions were halted at 15 min, the PC showed a Δ*D* ^–1^ of 18 μs, while a 40 min reaction yielded a Δ*D* ^–1^ of 108 μs (Figure 5a), reflecting increased amplicon accumulation over time. To access broader applicability, we generated a corresponding graph (Figure 5b) displaying Δ*D* ^–1^ values for biosensors exposed to positive LAMP amplicons from RSV and *Li*, alongside, the Δ*D* ^–1^ for NTC, derived by comparing the *D* ^–1^ of NTC-treated biosensors with that of untreated. All LAMP reactions used for this comparison were standardized at 20 min – chosen based on gel electrophoresis results (Figure 3) – to ensure sufficient amplification for biosensor detection in clinical scenarios where viral concentrations are unknown.

**Figure 5:**
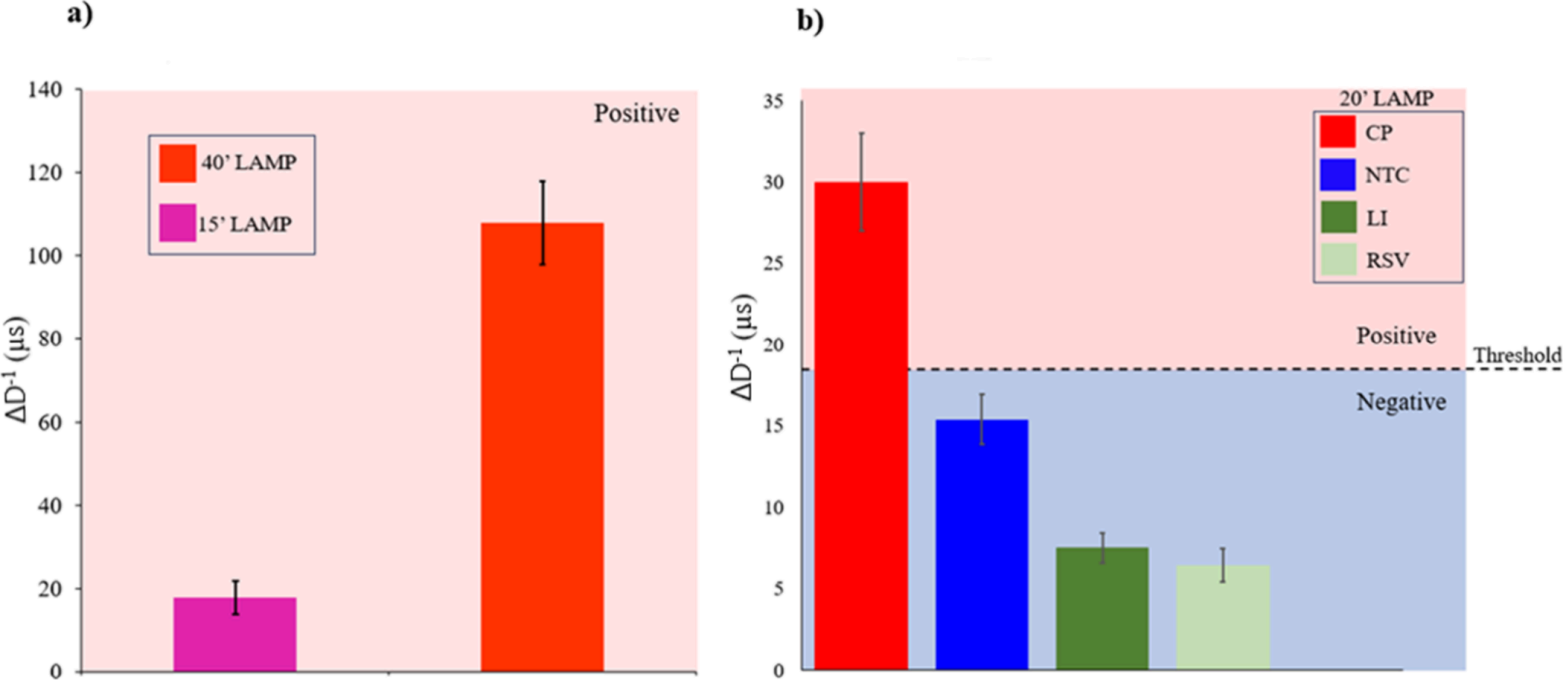
Biosensor titration analysis using DDLS, illustrating changes in diffusivity (Δ*D* ^–1^) following exposure to LAMP reaction ion products. (a) Δ*D* ^–1^ values for positive control (PC) samples after 15 min (18 μs) and 40 min (108 μs) of LAMP amplification. (b) Δ*D* ^–1^ values for biosensors exposed to LAMP amplicons from various pathogens, along with nontemplate control (NTC), after 20 min of amplification. Samples containing nonspecific genetic materials produced Δ*D* ^–1^ comparable to the NTC, confirming the specificity of the biosensor for its intended target. A threshold value above 20 μs (Δ*D* ^–1^) was established to differentiate positive from negative results. Error bars represent standard deviations from 10 replicate measurements.

Titration with nonspecific genetic material confirmed the specificity of the biosensors, as these samples produced Δ*D* ^–1^ values comparable to those of the NTC, and remained consistently below the values observed for PC samples. Notably, even when LAMP reactions halted at 15 min, the Δ*D* ^–1^ for PC samples exceeded the predefined threshold of 20 μs, established from 20 min standard reactions, indicating that diffusivity values above this threshold can reliably be interpreted as positive results.

### 3.5 Validating Biosensors with Clinical Samples

A set of nine qPCR-confirmed positive samples and nine negative samples, covering a range of cycle threshold values (Table 1) in qPCR were analyzed. A boxplot was generated using Python to visualize the distribution of results (Figure 6).

**Figure 6:**
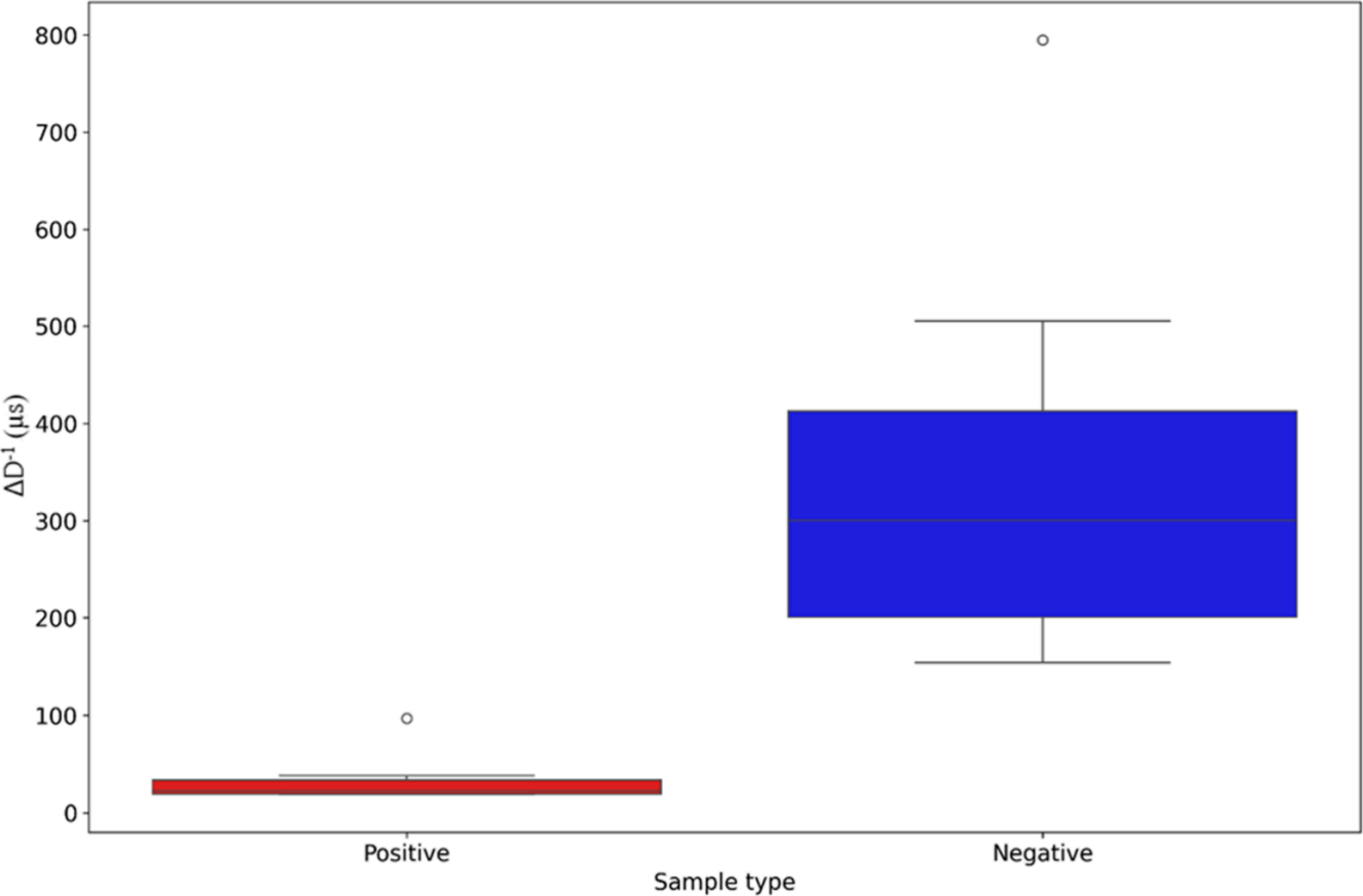
A box plot was generated in Python to illustrate the results of clinical sample testing, with positive (left, red) and negative (right, blue) samples. The analysis included nine qPCR-confirmed med positive samples and nine negative samples. A statistically significant difference between the two groups was observed using the nonparametric Mann–Whitney U test (*p* < 0.05). The effect size, calculated using Cohen’s *d*, was 2.183, indicating a large effect (*d* > 0.8) and a clear distinction between positive and negative samples. The white circles represent the outliers.

**Table 1:** Clinical Samples and Associated qPCR Ct Values and Viral Copy Number

sample	Ct value	viral copy number
1	21.25	69,000.00
2	21.24	69,000.00
3	22.70	28,000.00
4	23.06	21,000.00
5	23.23	18,000.00
6	26.27	2000.00
7	31.22	93.000
8	19.92	165,000.0
9	30.96	110.00

Regarding the statistical analysis, the group of positive samples did not follow a normal distribution according to the Shapiro-Wilk test (*p* = 0.14), while the negative group did (*p* = 0.026). Additionally, Levene’s test indicated a significant difference in variance between the groups (*p* = 0.002). Given these findings, the null hypothesis *H*_0_: *D* ^–1^_p_ = *D* ^–1^_n_ (i.e., no difference in diffusivity between positive and negative groups) was tested using the nonparametric Mann–Whitney U test, which revealed a statistically significant difference (*p* < 0.05). The effect size, calculated using Cohen’s *d*, was 2.183, indicating a large effect (*d* > 0.8) and confirming a substantial distinction between the two groups. We conducted this test with 18 clinical samples. However, we acknowledge that increasing the sample size in future studies would be beneficial for more robust and reliable results. Viral copy numbers corresponding to the Ct values of each clinical sample were determined based on a calibration curve, as described by Alves et al.^15^ and are presented in Table 1.

## 4 Discussion

TEM images and UV–vis spectroscopy measurements conducted in this study confirmed the successful synthesis of AuNRs (Figure 1) using a cost-effective and straightforward chemical method.^20^ AuNRs bioconjugation was characterized using UV–vis spectroscopy (Figure 2), which indicated that molecular probes specifically designed to bind to the viral target sequence were bound to GNRs.

Regarding the LAMP test for the diagnosis of SARS-CoV-2, Figure 3 shows samples subjected to the LAMP reaction for different incubation times, highlighting both their color patterns and the corresponding band profiles on the agarose gel. It is important to note that the sample incubated for only 15 min did not display any visible bands on the gel; however, it still contained sufficient genetic material to yield a positive result in our test, demonstrating the high sensitivity of the method. Another noteworthy observation is the sample incubated for 30 min, which exhibited an orange coloration rather than the expected yellow. This finding underscores the importance of complementary analytical approaches that can overcome the inherent ambiguity of color-based LAMP detection.

UV–Vis spectroscopy is a conventional analytical parameter that allows us to evaluate the efficiency of our test using an alternative method, independent of DDLS measurements. Figure 4 shows that the absorbance spectra of sensors exposed to the positive control (PC) remain stable, displaying the characteristic two-band profile corresponding to the transverse and longitudinal plasmon modes. In contrast, the spectra of samples exposed to the no-template control (NTC) indicate partial disintegration of the nanoparticles. This pattern of sensors behavior upon exposure to the PC and NTC is consistent with previously published data.^24,25^ Another important factor is the difference in color observed in the wells between positive and negative samples. This distinction serves as an additional and visually detectable parameter that clearly differentiates the two types of samples.

In our system, careful adjustment of the biosensor medium was crucial for maintaining consistent behavior between positive and negative samples. We introduced DMSO to enhance probe–analyte interaction^26,27^ and a custom cleanser, bare gold nanorods with LSPR (850 nm) - non resonant with the DDLS wavelength, aimed to reduce the amount of interfering particles present in nasopharyngeal samples. The presence of complex matrix components of the clinical samples can induce aggregation of nanosensors through unstable and nonspecific interactions between certain compounds—typically proteins—and the bioconjugated molecules. Such interactions generate background noise in the DDLS signal, leading to decreased sensitivity and measurement accuracy. In this study, we use custom cleansers to minimize such interferences through its adsorption onto the positively charged CTAB-capped lateral surfaces of the AuNRs. The non resonant GNRs are added to the samples and effectively eliminates potential interfering components through a passive “cleansing” mechanism, thereby enhancing both signal quality and analytical reliability.

In DDLS analysis, the primary parameter to be evaluated is the alteration in diffusivity. The tests using the positive control (PC) and the no-template control (NTC) were performed in a clean system (Figure 5), where PC consisted of a LAMP reaction using RNA extracted from SARS-CoV-2 cultivated in cell culture and NTC used ultrapure water as the sample. In this clean system, the PC exhibited higher changes in diffusivity (Δ*D* ^–1^) when compared to the NTC. This phenomenon can be attributed to the hybridization between the LAMP amplicons of PC samples and their corresponding probe functionalized onto the biosensor surface. Such hybridization results in the formation of double-stranded DNA bound to the sensor, increasing its hydrodynamic size and steric volume, and consequently changing its diffusivity relative to the unbound sensor. Conversely, in NTC within this clean system, significant smaller changes in sensor diffusivity were observed. A similar trend can be observed in Figure 5B, where amplicons from two different species were introduced into the system.

Regarding clinical samples, boxplot analysis of the samples (Figure 6) confirmed a clear separation between positive and negative groups. However, in this system, residual components from the buffer in which the nasopharyngeal swab was deposited (VTM) remain present in the RNA extracted from these samples and negative samples induced very pronounced alterations in biosensor’s diffusivity, suggesting the occurrence of aggregation. Nonspecific bindings remain a persistent problem that adversely affects the performance of biosensors.^28^ In our study, this phenomenon can be associated with the presence of guanidine isothiocyanate in the viral transport medium and RNA extraction buffers,^29^ which exhibits strong affinity for gold surfaces and may promotes biosensor destabilization and aggregation through the adsorption of thiocyanate ions onto the lateral surfaces of the AuNRs.^30^ This interpretation is reinforced by the pronounced drop in the scattering intensity—a secondary parameter that can also be monitored in our setup and is directly related to the number of biosensors dispersed in solution. Biosensors exposed to positive clinical samples presented much smaller alterations in diffusivity, indicating that the hybridization of LAMP amplicons with their corresponding probes leads to sensor stabilization, possibly due to a steric effect. This steric protection might come from the LAMP amplicons bound to the biosensor’ probes. LAMP amplicons are long molecules that readily form secondary structures, allowing them to shield GNR’s lateral surfaces. These characteristics of the nanosensor–amplicon interaction help maintain the diffusivity alterations of positive clinical samples at levels similar to those observed in the control system, typically below 50 μs.

In terms of sensitivity, the clinical sample with the highest Ct value corresponded to 110 viral copies/μL, consistent with previously reported rapid tests.^31,32^ This value is likely an underestimate, as samples with even higher Ct values were not assessed, suggesting that the true sensitivity of our assay may exceed 100 copies/μL. By comparison, a 40 min LAMP assay using the same primers achieved a Limit of Detection (LoD) of 19 copies/μL with serially diluted positive samples.^15^ These results indicate that, even with a 20 min runtime and without testing samples with higher Ct values, our assay demonstrates strong and promising sensitivity.

In summary, the integration of AuNR-based biosensors with DDLS provides a robust, adaptable, and sensitive platform for nucleic acid detection. This represents a significant advancement in the development of novel diagnostic tools. Importantly, it overcomes a major limitation of LAMP-false positives caused by nonspecific amplification typically detected via pH indicators. The platform is not only effective for SARS-CoV-2 but also offers potential for expansion to other pathogens and biomolecular targets. The ability to fine-tune system parameters and select appropriate readout strategies makes this a powerful tool for rapid, field-deployable diagnostics and improved disease control.

## 5 Conclusions

The research presented in this study introduces a promising platform for optimizing molecular detection in LAMP-based diagnostics. The findings highlight the remarkable potential of gold nanorods as biosensors to enhance LAMP performance by addressing one of its major limitations – false positives caused by nonspecific amplification. This novel approach enables direct detection of amplification products and has successfully reduced a COVID LAMP reaction time from 40 min, with optimal performance observed within 20 min under controlled conditions. These results demonstrate the platform’s potential for rapid, accurate, and label-free pathogen identification. We emphasize that further validation is necessary before definitive claims of selectivity and sensitivity can be established.

## Supplementary Material



## Notes

The sequences of the LAMP primers used in the experiments and the reference to the study from which these sequences were obtained (PDF)
