# Supplementary material for: Gold Nanorods Enable Rapid and Label-Free SARS-CoV‑2 Detection through LAMP Assay
Source: ACS Omega. 2026 Jan 23;11(5):7359–66. doi: 10.1021/acsomega.5c08097 (PMC12902867; doi:10.1021/acsomega.5c08097)
Supplement: Supplementary file 1 [file ao5c08097_si_001.pdf]

Gold Nanorods Enable Rapid and Label-Free SARS-CoV-2 Detection Through LAMP Assay

Amanda Bonoto Gonçalves[1,3]†, Iara Apolinário Borges[3]†, Kennedy Batista Gonçalves[2,3], Caroline Magalhães Junqueira[2,3], Letícia Trindade Almeida[1], Rosimeire Coura Barcelos[4], Pedro Augusto Alves[1], Lívia Siman Gomes[2,3*], Rubens Lima do Monte Neto[1]‡ & Anna Carolina Pinheiro Lage[1]‡

†These authors contributed equally to this work.

‡These authors jointly supervised this work.

Affiliations

[1]Grupo de Pesquisas em Biotecnologia Aplicada ao Estudo de Patógenos (BAP) - Instituto René Rachou, Fundação Oswaldo Cruz, IRR/Fiocruz Minas, Av. Augusto de Lima, 1715, Belo Horizonte, 30190-009, Minas Gerais, Brazil.

[2]Departamento de Física, Instituto de Ciências Exatas, Universidade Federal de Minas Gerais, Av. Presidente Antônio Carlos 6627, Pampulha, 31270-901, Belo Horizonte, Brazil.

[3]Centro de Tecnologia em Nanomateriais e Grafeno da Universidade Federal de Minas Gerais, CTNano – UFMG, Rua Professor José Vieira de Mendonça 520, Engenho Nogueira, 31310-260, Belo Horizonte, Brazil.

[4]Universidade Federal De São João Del Rei, Campus Centro Oeste Dona Lindu, Rua Sebastião Gonçalves Coelho, 400, Chanadour, 35501-296, Divinópolis/MG, Brazil.

*Corresponding Authors: liviasg@fisica.ufmg.br.

Supporting Information

Table S1: Nucleotide sequences of LAMP primers

Primer	Nucleotide Sequence	Reference
N_Set2_F3	TGGACCCCAAAATCAGCG	Alves *at el* 2021
N_Set2_B3	GCCTTGTCCTCGAGGGAAT	
N_Set2_FIP	CCACTGCGTTCTCCATTCTGGTAA ATGCACCCCGCATTACG	
N_Set2_BIP	CGCGATCAAAACAACGTCGGCCC TTGCCATGTTGAGTGAGA	
N_Set2_LF	TTGAATCTGAGGGTCCACCAAA	
N_Set2_LB	GGTTTACCCAATAATACTGCGTCTT	
F3 - RSV	TGACATCAGAAATACAGTAAT	Ikuyo Takayama *et al* 2019
B3 - RSV	CGTTTTTTAAGATTGTTTGC	
FIP- RSV	CATCCCACGATGTGGAAGGAATGAAGTACAAAAATGC	
BIP- RSV	GCTGGCCTTGAACAATAATAGTCTAAATATCTGAAGACC	
LF- RSV	GAGCCACTTCCTCACCATTG	
LB- RSV	GAGCCACTTCCTCACCATTG	

*The *Leishmania infantum* set is under patent review and cannot be disclosed.

REFERENCES

1. Alves, P. A., Oliveira, E. G. De, Franco-luiz, A. P. M. & Almeida, L. T. Optimization and Clinical Validation of Colorimetric Reverse Transcription Loop-Mediated Isothermal Amplification , a Fast , Highly Sensitive and Specific COVID-19 Molecular Diagnostic Tool That Is Robust to Detect SARS-CoV-2 Variants of Concern. *Front Microbiol* 12, (2021)

2. Takayama I, Nakauchi M, Takahashi H, Oba K, Semba S, Kaida A, Kubo H, Saito S, Nagata S, Odagiri T, Kageyama T. Development of real-time fluorescent reverse transcription loop-mediated isothermal amplification assay with quenching primer for influenza virus and respiratory syncytial virus. J Virol Methods.267:53-58 (2019).
